# Role of prostate health index to predict Gleason score upgrading and high-risk prostate cancer in radical prostatectomy specimens

**DOI:** 10.1038/s41598-021-96993-2

**Published:** 2021-08-31

**Authors:** Hwanik Kim, Gyoohwan Jung, Jin Hyuck Kim, Seok-Soo Byun, Sung Kyu Hong

**Affiliations:** 1 Department of Urology, Seoul National University Bundang Hospital, Seongnam, South Korea; 2 Department of Urology, Seoul National University College of Medicine, Seoul, South Korea

**Keywords:** Cancer, Molecular biology, Biomarkers, Diseases, Molecular medicine, Oncology, Urology

## Abstract

We evaluated the role of prostate health index (PHI) in predicting Gleason score (GS) upgrading in International Society of Urological Pathology Grade Group (ISUP GG) 1 & 2 prostate cancer (PCa) or adverse pathologic outcomes at radical prostatectomy (RP). A total of 300 patients with prostate specific antigen ≥ 3 ng/mL, PHI and prostate biopsy (71 patients with RP included) were retrospectively included in the study. The primary study outcomes are PCa and clinically significant PCa (csPCa, defined as ISUP GG ≥ 2) diagnostic rate of PHI, and GS upgrading rate at RP specimen. The secondary outcomes are the comparison between GS upgrading and non-upgrading group, GS upgrading and high-risk PCa (ISUP GG ≥ 3 or ≥ pT3a) predictability of preoperative clinical factors. Overall, 139 (46.3%) and 92 (30.7%) were diagnosed with PCa and csPCa, respectively. GS upgrading rate was 34.3% in all patients with RP. Significant differences were shown in the total prostate volume (p = 0.047), the distribution of ISUP GG at biopsy (p = 0.001) and RP (p = 0.032), respectively. PHI values ≥ 55 [Odds ratio (OR): 3.64 (95% confidence interval (CI) = 1.05–12.68, p = 0.042] and presence of PI-RADS lesion ≥ 4 (OR: 7.03, 95% CI = 1.68–29.51, p = 0.018) were the significant predictors of GS upgrading in RP specimens (AUC = 0.737). PHI values ≥ 55 (OR: 9.05, 5% CI = 1.04–78.52, p = 0.046) is a significant factor for predicting adverse pathologic features in RP specimens (AUC = 0.781). PHI could predict GS upgrading in combination with PIRADS lesions ≥ 4 in ISUP GG 1 & 2. PHI alone could evaluate the possibility of high-risk PCa after surgery as well.

## Introduction

A major challenge in prostate cancer (PCa) treatment is to detect those which should not be managed with active surveillance (AS) ^1^. To overcome the limitations of total prostate-specific antigen (tPSA) for diagnosis, [−2]proPSA (p2PSA) derivatives, such as Prostate Health Index (PHI), percentage of p2PSA (%p2PSA) and PHI density (PHID) have been suggested. %p2PSA and PHI have been associated with improved overall detection and aggressive PCa detection over tPSA. and free PSA (fPSA)/tPSA ratio (%fPSA) in several studies ^2–4^.

Currently, no single biomarker has the ideal performance characteristics necessary for the detection and risk stratification of PCa. The PHI seems to be a simple and inexpensive test for a multivariant approach to PCa screening and management. The PHI improves cancer prediction at initial and extended biopsy stages and even has some capability to predict disease recurrence after radical prostatectomy (RP) ^3^. As the age-specific PCa incidence rates increase with age in Asian countries, better markers are needed to differentiate aggressive PCa from indolent, in order to better counsel patients as to appropriate treatment options ranging from radical treatment to AS ^4,5^. In this study, we aimed to evaluate the role of PHI in localized PCa and whether it can function as an independent predictor of Gleason score (GS) upgrading at RP specimen in GS 3 + 3 PCa, of unfavorable or high risk PCa, and of adverse pathologic features in RP specimens.

## Patients and methods

### Patient selection and evaluation

All patients with transrectal prostate biopsy, PSA ≥ 3 ng/mL, and clinical stage ≤ cT3aN0 between 04/2019 and 07/2020 were included in this retrospective cohort analysis. Among them, 139 (46.3%) had a histologically confirmed diagnosis of PCa from transrectal ultrasound guided biopsy (TRUS-Bx) or magnetic resonance imaging-guided biopsy (MRI-GB) within the 3 months before surgery. Due to the referral nature of our practice, genitourinary pathologists with more than 15 years of experience reviewed all the biopsy slides. Thus, the biopsy GS reported represents the result of our internal review, and detailed biopsy information was available for all patients. Prior to prostate biopsy, blood was drawn to measure the prebiopsy tPSA, fPSA, and p2PSA levels. Blood samples were processed using the Access 2 immunoassay kit (Beckman Coulter, Brea, CA, USA) ^6,7^.

RP was performed using a robot-assisted approach by experienced urologists. Pelvic lymph node dissection was carried out according to the operating surgeons’ preferences. Surgical specimens were processed and analyzed using a standardized technique by the same genitourinary pathologists who reviewed biopsy slides. Men with previous prostate surgery, active urinary tract infection or those using medications that affect PSA levels (e.g., 5-α reductase inhibitors) were excluded. The primary study outcomes were PCa and clinically significant PCa (csPCa) diagnostic rate of PHI and GS upgrading rate at RP specimen. The secondary study outcomes were significant clinical factors predicting GS upgrading or adverse pathologic features (International Society of Urological Pathology Grade Group [ISUP GG] ≥ 3 or ≥ pT3a) at RP specimen. The institutional review board approved the study (IRB number: B-2011-648-104). A written informed patient consent was waived by the Seoul National University Bundang Hospital Institutional Review Board due to the retrospective nature of study. All methods were conducted in accordance with relevant guidelines and regulations (the ethical standards of the 1964 Declaration of Helsinki and its later amendments or comparable ethical standards).

### Transrectal ultrasound guided biopsy and MRI guided biopsy

Transrectal prostate biopsies were obtained under local anesthesia using an automatic biopsy gun and an 18-G needle under TRUS guidance. In all, 12 cores (six in the peripheral zone and six in the transitional zone) were taken in all patients. In the case of MRI guided biopsy, at least two or more cognitive fusion-targeted or MRI/Ultrasound image fusion (BioJet MRI-Ultrasound Fusion system with bk5000 ultrasound, BK Medical, United States) biopsy cores were added for each lesion in patients with suspicious or equivocal lesions in mpMRI ^8^. Two uroradiologists with more than 10-years of experience including more than a thousand pelvic MRIs read, graded the level of suspicion for clinically significant cancer from mpMRI mapping images using the Prostate Imaging Reporting and Data System Version 2 (PIRADS V2) scale 1–5 ^9^.

### Pathological analysis

The same expert pathologists from Department of Pathology in our center assessed specimens from needle biopsies and RP. We followed the recommendations of the 2014 ISUP consensus for GS. The highest-grade pattern was recorded if there were multiple scores at multiple biopsy sites or multiple cancer nodules in the RP specimen. We excluded patients presenting minor tertiary pattern 5 on prostatectomy. GS upgrading was defined as any increased total sum in the pathological GS compared with that of the biopsy GS. In addition, an increase in the main structure score without a change in the total sum (ex. ISUP GG 2 → 3) was also defined as GS upgrading ^10^. Adverse pathologic feature was defined as non-organ-confined disease (pT3 or higher) or GS ≥ 4 + 3 (ISUP GG ≥ 3) after RP ^11,12^.

### Data collection

The clinical and biopsy variables included age at surgery, preoperative initial PSA series (tPSA, fPSA, p2PSA with the latest collected right before biopsy), clinical stage, primary and secondary (highest) Gleason grading on biopsy, number of positive cores, number of total cores and percentage of positive/total cores, maximum percentage of surface specimen tumor involvement, presence of perineural invasion, and/or high-grade prostatic intraepithelial neoplasia in biopsy specimens. Pathologic variables were primary and secondary (highest) GS, pT and pN stage, extraprostatic extension, seminal vesicle invasion, and lymph node metastasis. The American Joint Committee on Cancer TNM 8th edition (2018) was used for pathologic staging, and GS was assigned according to the 2014 ISUP modified Gleason scoring system ^13^.

fPSA and PHI percentages were calculated using the formulas:

1 $$\% {\text{freePSA }} = \, \left( {{\text{fPSA}}/{\text{tPSA}}} \right) \, \times { 1}00,$$

2 $${\text{PHI }} = \, \left( {{\text{p2PSA}}/{\text{fPSA}}} \right){\text{ x}}\sqrt {{\text{tPSA}}}$$

CsPCa was defined as the presence of at least one sample with a Gleason four or five grade lesion, or ISUP GG ≥ 2.

### Statistical analysis

In addition to descriptive statistics, we used the chi-square test for comparing categorical variables and the Students t test, Wilcoxon rank sum test, Mann Whitney U-test, or Kruskal–Wallis test for comparison of continuous variables. Clinical data and detailed biopsy information were analyzed in multivariable prediction models using logistic regression ^14^. The univariate and multivariate logistic regression analyses were performed to detect risk factors for GS upgrading in ISUP GG 1 & 2, adverse pathologic features at all RP specimens. Multivariate analysis using variables with a p value < 0.05 in univariate analysis was performed to identify which variables were independently predictive of outcomes (GS upgrading and adverse pathologic features). The predictive models’ accuracy was compared using area under the receiver operating curve (AUC). All tests were two sided with a value of 0.05. The statistical analysis was performed using IBM Statistical Package for the Social Science Statistics for Windows (SPSS) version 22.0 (IBM Corp. Released 2013. IBM SPSS Statistics for Windows, Version 22.0. Armonk, NY, USA https://www.ibm.com/products/spss-statistics).

### Ethical statements

The study was conducted according to the guidelines of the Declaration of Helsinki, and approved by the Institutional Review Board of Seoul National University Bundang Hospital (B-2011-648-104 and date of approval: 9th/November/2020).

## Results

### Patients

Of 300 patients who were evaluated for PHI and had prostate biopsy, 92 (30.7%) were diagnosed with csPCa. The baseline characteristics according to biopsy outcomes are summarized in supplementary Table 1. There were significant differences in PSA level (p = 0.014), PSA density (PSAD) (p = 0.002), PHID (p < 0.001), cancer core number at prostate biopsy (p = 0.001), cancer positive core rate (p < 0.001), PCa detection rate (p < 0.001), csPCa detection rate (p < 0.001), distribution of ISUP GG at biopsy (p < 0.001), and distribution of ISUP GG (p < 0.001) at RP among groups if classified by PHI level range (0–26.9/27.0–35.9/36.0–54.9/ ≥ 55.0).

### Baseline characteristics according to Gleason score (GS) upgrading in RP specimens

Subgroup analysis, classified by GS upgrading, was performed and is described in Table 1. 71 patients underwent RP and GS upgrading rate was 33.8% (24/71) in all patients with RP. Significant differences were shown in the preoperative prostate volume (p = 0.014), the distribution of ISUP GG at biopsy (p = 0.001), and the distribution of ISUP GG (p = 0.016) at RP pathology.

**Table 1:** Baseline characteristics according to Gleason score (GS) upgrading in RP specimens.

Variable	GS non-upgrading (n = 47)	GS upgrading (n = 24)	p value
Age (years)	68.0 (62.0–70.0)	71.0 (61.0–72.5)	0.249
BMI	25.4 (24.4–27.8)	24.9 (24.3–27.0)	0.767
PSA (ng/mL)	7.3 (5.5–10.3)	9.5 (4.8–19.3)	0.133
PHI	84.0 (49.6–93.9)	61.3 (48.7–125.8)	0.402
PHI density	2.05 (1.50–3.00)	1.99 (0.92–3.47)	0.755
Total prostate volume (cc)	32.9 (28.2–40.0)	38.0 (31.4–51.2)	0.047
Number of cancer core	6.0 (4.0–7.8)	5.0 (2.0–8.0)	0.066
**ISUP grade group at biopsy**			0.001
1	2 (4.3%)	10 (41.7%)	
2	17 (36.2%)	8 (33.3%)	
3	17 (36.2%)	3 (12.5%)	
4	11 (23.4%)	3 (12.5%)	
**PHI group**			0.574
2	1 (2.1%)	1 (5.3%)	
3	14 (29.8%)	8 (33.3%)	
4	32 (68.1%)	15 (62.5%)	
**ISUP grade group at RP**			0.032
1	1 (2.1%)	0	
2	24 (51.1%)	10 (41.7%)	
3	18 (38.3%)	8 (33.3%)	
4	4 (8.5%)	2 (8.3%)	
5	0	4 (16.7%)	
**GS at RP**			0.010
6	1 (2.2%)	0	
7	42 (89.4%)	18 (75.0%)	
8	4 (8.5%)	2 (8.3%)	
9	0	4 (16.7%)	
≥ pT3a	14 (29.8%)	10 (41.7%)	0.317
EPE at MRI	9 (32.1%)	2 (14.3%)	0.215
CS lesion at MRI	26 (55.3%)	12 (50.0%)	0.671
**PIRADS score**			0.505
3	2/28 (7.1%)	1/13 (7.7%)	
4	17/28 (60.7%)	6/13 (46.2%)	
5	9/28 (32.1%)	6/13 (46.2%)	

Values are presented as median (interquartile range) or number (%).

*BMI* body mass index, *CS* lesion: clinically significant PIRADS lesion, *EPE* extraprostatic extension, *GS* Gleason score, *ISUP* International Society of Urological Pathology, *MRI* magnetic resonance imaging, *PHI* prostate health index, *PIRADS* prostate imaging reporting and data system, *PSA* prostate-specific antigen, *RP* radical prostatectomy.

### Predictor of GS upgrading at RP

24 patients with RP had GS upgrading at RP specimen. Multivariate logistic regression analysis (Table 2) revealed PHI values ≥ 55 (Odds ratio (OR): 3.64 [95% Confidence interval [CI] = 1.05–12.68, p = 0.042) and presence of PI-RADS lesion ≥ 4 (OR: 7.03, 95% CI = 1.68–29.51, p = 0.018) were the significant predictors of GS upgrading in RP specimens (AUC = 0.737).

**Table 2:** Logistic regression analysis for predicting Gleason score upgrading at RP.

Variable	Univariate			Multivariate		
	OR	95% CI	p value	OR	95% CI	p value
Age (years)	1.04	0.97–1.12	0.247			
Initial prebiopsy PSA (ng/mL)	1.08	0.99–1.18	0.096			
PSA density ≥ 0.15	0.71	0.24–2.07	0.525			
Positive core rate ≥ 50%	0.78	0.28–2.19	0.634			
Total prostate volume (cc)	1.04	1.00–1.09	0.068			
PHI ≥ 55	7.17	1.78–28.93	0.006	3.64	1.05–12.68	0.042
PHI density	0.94	0.62–1.41	0.751			
MRI targeted prostate biopsy	1.20	0.45–3.25	0.716			
Clinically significant PIRADS lesion ≥ 4	4.44	1.37–14.45	0.013	7.03	1.68–29.51	0.018

*OR* odds ratio, *CI* confidence interval, *PSA* prostate specific antigen, *PHI* prostate health index, *MRI* magnetic resonance imaging, *PIRADS* prostate imaging reporting and data system, *ISUP GG* International Society of Urological Pathology grade group.

### Predictor of adverse pathologic features at RP (adverse pathologic features: ≥ pT3a or GS ≥ 4 + 3)

13 patients had adverse pathologic features at RP. PHI values ≥ 55 (OR: 9.05, 5% CI = 1.04–78.52, p = 0.046) is a significant factor for predicting adverse pathologic features in RP specimens (Table 3) (AUC = 0.781).

**Table 3:** Logistic regression analysis for predicting adverse pathologic features at RP (adverse pathologic features: ≥ pT3a or Gleason score ≥ 4 + 3).

Variable	Univariate			Multivariate		
	OR	95% CI	p value	OR	95% CI	p value
Age (years)	1.03	0.99–1.07	0.102			
Initial prebiopsy PSA (ng/mL)	1.01	0.99–1.04	0.352			
PSA density ≥ 0.15	9.42	4.04–21.98	< 0.001	4.93	0.55–44.19	0.154
Prostate Health Index (PHI)	1.009	1.002–1.069	0.011			
PHI ≥ 55	5.18	2.32–11.58	< 0.001	9.05	1.04–78.52	0.046
PHI density	1.75	1.37–2.22	< 0.001			
MRI targeted prostate Biobio	0.72	0.37–1.38	0.321			
Extraprostatic extension in MRI	22.90	6.37–82.26	< 0.001	2.97	0.78–11.38	0.112

*OR* odds ratio, *CI* confidence interval, *PSA* prostate specific antigen, *MRI* magnetic resonance imaging, *PIRADS* prostate imaging reporting and data system.

## Discussion

Although numerous studies on PHI have been reported, relatively little has been revealed about whether PHI can predict high-risk PCa. This might be due to recent trends in which PHI has been used to gain more certainty on PCa for people with grey zone PSA levels, and the relatively low probability that high-risk PCas are actually detected in the PSA grey zone. Based on the fact that biopsy has a significant role in the diagnosis and staging of PCa ^15^, AS has recently been seen as an option for low-risk groups of patients. However, selection criteria are not yet definitively established ^16–18^. Tsang et al*.* reported the necessity of PSAD for AS and a cutoff for PSAD in identifying adverse pathological outcomes in an Asian cohort. They concluded that PSAD with a cutoff at 0.19 ng/ml/ml provided the best balance between sensitivity and specificity in predicting adverse pathological disease ^19^. Compared to that study, our results showed that a PSAD level ≥ 0.15 ng/ml/ml could also significantly predict adverse pathologic features at RP (OR: 7.32, 95% CI = 1.19–45.08, p = 0.032).

A large-scale study reported the frequency of GS upgrading and downgrading in 7643 patients were 36.3 and 12.0%, respectively, revealing a stronger tendency for biopsies to underestimate rather than overestimate the true GS ^20^. Detection of GS upgrading at RP specimen is fundamental. It may potentially lead to reduction of probability for undertreatment of undergraded patients at the initial biopsy, or has been associated with adverse pathological outcomes, such as positive surgical margin status and biochemical recurrence (BCR) ^21,22^. GS upgrading can help predict those high risk patients that should not be managed with AS. Imnadze et al. demonstrated that adverse pathologic features at RP are associated with an increased BCR risk which is influenced substantially by pretreatment factors, and pathologic features in isolation cannot dictate treatment ^23^. For counseling a patient who is considering AS, clinicians should share meaningful information about chances of having disease with adverse pathology, and the oncologic risk associated with such findings in the specific context of preoperative risk including PHI results. Furthermore, PHI results might also be used to aid a clinician in the selection of high PHI with ISUP GG ≥ 3 patients, if upgraded at RP specimen, for post-RP adjuvant therapy.

From our multivariate analysis, the combination of PHI and mpMRI is better at predicting GS upgrading than mpMRI alone (Table 2). Although PHI, (considered as a new medical technology in South Korea), is not yet reimbursed by national health insurance, a combination of PHI and MRI-GB can be strongly recommended for patients with PSA grey zone levels, to increase the diagnostic rate of PCa and csPCa. It is believed that the insurance drawback can be overcome through clear communication during consultations, including stressing the need for PHI to patients retaining private insurance. In one study, the PHI also improved the cost-effectiveness of PCa detection with a 17% reduction in costs of diagnosis and a 1% reduction in the total costs for treatment of PCa. These cost savings were due to a reduction in the number of unnecessary biopsies ^17^. Another study ^24^ reported the use of the PHI in the PCa detection (19.1% diagnosed as PCa) in 157 Asian males with a tPSA of 4–10 ng/mL. At a PHI cutoff level of 55, 42.9% of patients had PCa, and all of them were at GS > 6. The PCa detection rate was 55.5% in our study, which was higher than theirs, and use of PHI demonstrated superior performance over PSA in PCa detection (AUC—PHI: 0.672 vs tPSA: 0.594) ^24^.

In this study, adverse pathology definition was T3a or higher or ISUP GG 3, to confirm that PHI can significantly predict adverse pathology. Though varying definitions of adverse pathology exist, the criteria defined by either primary Gleason pattern ≥ 4 or pT3-4 disease, like ours, appears to most accurately predict adverse pathology or BCR in patients with lower risk PCa at the time of diagnosis ^25^.

The limitation of this study is that it is a retrospective design with a small sample size and a single ethnicity. Our sample size might be considered too low to solve the main question of the study. Since AS can be considered as a first treatment option for low-grade PCa, there may be a non-negligible selection bias in our study. In fact, in South Korea, where PHI is not yet reimbursed by national insurance, the total number of PHI tests are not high, as it was initiated less than five years ago and there is scarce long-term follow-up data. In addition, csPCa as defined in this study does not have a standard global definition, which may make it difficult to apply in real-world clinical practice. Our predictive model may be considered as a model that analyzes the association between PHI and high-risk PCa rather than formally evaluating the predictability of total PCa. Another limitation is that clinicians need to be cautious in applying our results in real world practice considering that GS upgrading at prostatectomy may instead indicate under-scoring at biopsy. Finally, it was not possible to generate a comparative analysis with other available blood biomarkers, including PCA3 and genetic markers. In the near future, these limitations are expected to be overcome to some extent if a multicenter prospective randomized controlled trial is implemented. Nevertheless, this study is the first to provide clues that PHI can predict simultaneously GS upgrading in potential active surveillance candidates and high-risk PCa even from a small Korean cohort, and shows that PHI is reliable and can be added to existing evaluation tools for PCa diagnosis and prediction. The important clinical question in our study is whether PHI or PHI density improves upon PSA/PSA density in predicting GS upgrading or adverse pathology. Though with small numbered group and PHI analyzed as dichotomous variable, multivariate analysis showed relatively more associations of PHI with those features than PSA or PSAD. Moreover, relevant data for Koreans are scarce. Therefore, our results are worth applying to Korean all over the world and also satisfy unmet needs for clinicians counseling them. In addition, our results are expected to be utilized as supportive evidence for PCa patients who need radical treatments such as surgery or radiation therapy while potentially preventing excessive prostate biopsy.

## Conclusion

PHI could predict GS upgrading in combination with PIRADS lesions ≥ 4 in ISUP GG 1 & 2. PHI alone could evaluate the possibility of unfavorable or high-risk PCa with adverse pathologic features after surgery as well. PHI appears to improve csPCa detection and provide prognostic value. These results provide an opportunity to define appropriate treatment strategies for AS candidates and also can be of significant assistance for the pre-treatment counseling of all potential PCa patients. Our study findings could be helpful for the management of both patients in AS and patients considered for RP. Further studies are still needed on the predictiveness of PHI on several aspects on PCa in the future.

## Supplementary Information

Supplementary Table S1.
