# Supplementary material for: Role of prostate health index to predict Gleason score upgrading and high-risk prostate cancer in radical prostatectomy specimens
Source: Sci Rep. 2021 Aug 31;11:17447. doi: 10.1038/s41598-021-96993-2 (PMC8408259; doi:10.1038/s41598-021-96993-2)
Supplement: Supplementary file 1 — Supplementary Table S1. [file 41598_2021_96993_MOESM1_ESM.docx]

Supplementary Material

Supplementary Table 1. Baseline characteristics according to biopsy outcomes

Supplementary Table 1. Baseline characteristics according to biopsy outcomes

| Variable | Presence of prostate cancer (Pca) | | | Presence of clinically significant PCa (csPCa) | | |
| --- | --- | --- | --- | --- | --- | --- |
|  | No PCa  (n=161) | Total PCa  (n=139) | p value | non-csPCa  (n=47) | csPCa  (n=92) | p value |
| Age (y) | 61.2 ± 10.7 | 66.7 ± 8.4 | <0.001 | 65.6 ± 8.3 | 67.3 ± 8.5 | 0.245 |
| BMI (kg/m2) | 25.1 ± 2.7 | 25.6 ± 3.0 | 0.354 | 25.0 ± 2.6 | 26.0 ± 3.2 | 0.204 |
| PSA (ng/mL) | 6.87 ± 3.65 | 10.09 ± 13.00 | 0.005 | 7.21 ± 4.81 | 11.57 ± 15.43 | 0.014 |
| PSAD | 0.14 ± 0.08 | 0.25 ± 0.32 | <0.001 | 0.15 ± 0.09 | 0.30 ± 0.37 | <0.001 |
| PHI | 55.18 ± 26.27 | 76.68 ± 57.45 | <0.001 | 61.98 ± 43.03 | 84.19 ± 62.46 | 0.031 |
| PHID | 1.32 ± 0.88 | 2.18 ± 1.53 | <0.001 | 1.63 ± 1.54 | 2.46 ± 1.46 | 0.003 |
| Free PSA | 0.89 ± 0.67 | 1.12 ± 1.03 | 0.022 | 1.05 ± 0.49 | 1.16 ± 1.21 | 0.557 |
| P2PSA | 19.28 ± 14.60 | 32.79 ± 63.19 | 0.015 | 26.89 ± 28.18 | 35.80 ± 75.02 | 0.434 |
| %free PSA | 15.31 ± 7.29 | 14.57 ± 7.56 | 0.392 | 17.81 ± 7.14 | 12.92 ± 7.26 | <0.001 |
| Prostate size (mL) | 49.1 ± 22.6 | 40.6 ± 16.3 | <0.001 | 48.7 ± 20.6 | 36.4 ± 11.6 | <0.001 |
| Total biopsy core number | 13.4 ± 1.0 | 13.1 ± 1.3 | 0.024 | 13.4 ± 1.0 | 12.9 ± 1.4 | 0.024 |
| Number of cancer core | 0 | 4.1 ± 2.9 | <0.001 | 2.3 ± 1.7 | 5.1 ± 2.9 | <0.001 |
| Target biopsy core number | 1.4 ± 0.9 | 1.2 ± 1.0 | 0.026 | 1.5 ± 1.0 | 1.1 ± 1.0 | 0.025 |
| positive core rate (%) | 0 | 31.8 ± 22.8 | <0.001 | 16.8 ± 12.4 | 39.4 ± 23.1 | <0.001 |
| MRI findings (n=203) |  |  |  |  |  |  |
| ≥ PIRADS 4 lesion | 17 (14.5%) | 59 (68.6%) | <0.001 | 18 (38.3%) | 41 (44.6%) | 0.479 |
| EPE | 1 (0.9%) | 12 (14.0%) | <0.001 | 1 (2.7%) | 11 (22.4%) | 0.128 |
| Biopsy method |  |  | 0.126 |  |  | 0.008 |
| Conventional | 49 (30.4%) | 54 (38.8%) |  | 11 (23.4%) | 43 (46.7%) |  |
| MRI-fusion | 112 (69.6%) | 85 (61.2%) |  | 36 (76.6%) | 49 (53.3%) |  |
| Biopsy times |  |  | 0.115 |  |  | 0.003 |
| Initial | 110 (68.3%) | 110 (79.1%) |  | 30 (63.8%) | 80 (87.0%) |  |
| Repeat | 51 (31.7%) | 29 (20.9%) |  | 17 (36.2%) | 12 (13.0%) |  |
| ISUP Grade group  at Biopsy (n=139) |  |  |  |  |  | <0.001 |
| 1 |  | 47 (33.8%) |  | 47 | 0 |  |
| 2 |  | 38 (27.3%) |  | 0 | 38 (41.3%) |  |
| 3 |  | 32 (23.0%) |  | 0 | 32 (34.8%) |  |
| 4 |  | 20 (14.4%) |  | 0 | 20 (21.7%) |  |
| 5 |  | 2 (1.4%) |  | 0 | 2 (2.2%) |  |
| ISUP Grade group  at RP specimen (n=71) |  |  |  |  |  | <0.001 |
| 1 |  |  |  | 1 (8.3%) | 0 |  |
| 2 |  |  |  | 11 (91.7%) | 23 (39.0%) |  |
| 3 |  |  |  | 0 | 26 (44.1%) |  |
| 4 |  |  |  | 0 | 6 (10.2%) |  |
| 5 |  |  |  | 0 | 4 (6.8%) |  |
| GS upgrading rate |  |  |  | 11 (91.7%) | 14 (23.7%) | <0.001 |

Values are presented as mean±standard deviation, or number (%).

BMI: body mass index; CsPCa: clinically significant prostate cancer (ISUP GG 2 or over); EPE: extraprostatic extension; GS: Gleason score; ISUP: International Society of Urological Pathology; MRI: magnetic resonance imaging; PHI: prostate health index; PIRADS: Prostate Imaging Reporting and Data System; PSA: prostate specific antigen; RP: radical prostatectomy
